# The anti-inflammatory role of zDHHC23 through the promotion of macrophage M2 polarization and macrophage necroptosis in large yellow croaker (*Larimichthys crocea*)

**DOI:** 10.3389/fimmu.2024.1401626

**Published:** 2024-05-29

**Authors:** Ting Dai, Ziyue Zhao, Tingfang Zhu, Chenjie Fei, Li Nie, Jiong Chen

**Affiliations:** ^1^ State Key Laboratory for Managing Biotic and Chemical Threats to the Quality and Safety of Agro-products, Ningbo University, Ningbo, China; ^2^ Laboratory of Biochemistry and Molecular Biology, School of Marine Sciences, Ningbo University, Ningbo, China; ^3^ Zhejiang Key Laboratory of Marine Bioengineering, Ningbo University, Ningbo, China

**Keywords:** LczDHHC23, immune regulation, macrophage polarization, necroptosis, large yellow croaker

## Abstract

Zinc finger Asp-His-His-Cys motif-containing (zDHHC) proteins, known for their palmitoyltransferase (PAT) activity, play crucial roles in diverse cellular processes, including immune regulation. However, their non-palmitoyltransferase immunomodulatory functions and involvement in teleost immune responses remain underexplored. In this study, we systematically characterized the zDHHC family in the large yellow croaker (*Larimichthys crocea*), identifying 22 members. Phylogenetic analysis unveiled that each of the 22 *Lc*zDHHCs formed distinct clusters with their orthologues from other teleost species. Furthermore, all *Lc*zDHHCs exhibited a highly conserved DHHC domain, as confirmed by tertiary structure prediction. Notably, *Lc*zDHHC23 exhibited the most pronounced upregulation following *Pseudomonas plecoglossicida* (*P. plecoglossicida*) infection of macrophage/monocyte cells (MO/MΦ). Silencing *Lc*zDHHC23 led to heightened pro-inflammatory cytokine expression and diminished anti-inflammatory cytokine levels in MO/MΦ during infection, indicating its anti-inflammatory role. Functionally, *Lc*zDHHC23 facilitated M2-type macrophage polarization, as evidenced by a significant skewing of MO/MΦ towards the pro-inflammatory M1 phenotype upon *Lc*zDHHC23 knockdown, along with the inhibition of MO/MΦ necroptosis induced by *P. plecoglossicida* infection. These findings highlight the non-PAT immunomodulatory function of *Lc*zDHHC23 in teleost immune regulation, broadening our understanding of zDHHC proteins in host-pathogen interactions, suggesting *Lc*zDHHC23 as a potential therapeutic target for immune modulation in aquatic species.

## 1 Introduction

The zinc finger Asp-His-His-Cys motif-containing (zDHHC) proteins constitute a family of palmitoyltransferases (PATs) primarily tasked with catalyzing the addition of palmitate moieties onto protein substrates, a process known as S-palmitoylation (1). To date, 24 zDHHC members have been identified in mammalian species, with 23 in humans (zDHHC1–9, 11–24) and 24 in mice (zDHHC1–9, 11–25) (1). All of these members are characterized by the presence of a 50-residue long DHHC (Asp-His-His-Cys) domain, which serves as the active center for PATs (2). S-palmitoylation, the reversible attachment of palmitate to cysteine residues via thioester bonds, plays crucial roles in regulating protein membrane localization, trafficking, stability, and function (3). As integral players in protein S-palmitoylation, zDHHCs hence contribute significantly to the modulation of diverse cellular processes, including signal transduction, membrane dynamics, and protein-protein interactions (4). Recently, research has focused on the role of zDHHCs in modulating immune responses. Molecules involved in innate immunity, such as the pattern recognition receptor TLR2 (Toll-like receptor 2), the cytoplasmic DNA receptor cGAS (cyclic GMP-AMP synthase), the cytoplasmic bacterial peptidoglycan receptor NOD1 and NOD2 (nucleotide-binding oligomerization domain-containing protein 1/2), the type I interferon receptor IFNAR (interferon alpha/beta receptor), the transcription factor STAT3 (signal transducer and activator of transcription 3), and the cytokine TNF-α, have all been reported to undergo palmitoylation by various zDHHCs (5–9). Moreover, zDHHCs participate in the palmitoylation of adaptive immune molecules, such as CD4, CD8, and LAT (the inker for activation of T cells), which are associated with T cell function (10–12). Additionally, molecules involved in B cell function, like CD81, LAB (the linker for activation of B cells), were also palmitoylated by zDHHCs (13, 14). Despite primarily functioning as PAT for immune-related molecules, the non-PAT roles of zDHHCs in immunity are largely unexplored. Only zDHHC1 and zDHHC11 has been identified as a positive regulator of DNA virus-triggered signaling via STING interaction, independent of their PAT activity (15, 16). Therefore, investigating the non-PAT functions of the zDHHC family is a highly valuable area for further research.

To date, our understanding of the composition and function of the zDHHC family in lower species, including teleosts, remains limited. Systematic studies are needed to determine the number of zDHHCs present in teleosts and their respective functions, as well as to investigate the evolutionary clues of this family. Only five zDHHCs (zDHHC1, 13, 15, 16, and 17) have been reported and characterized for their functions in teleosts, with studies focusing mainly on zebrafish (zDHHC13, 15, 16, and 17) (17–20). Independently of their PAT activity, zebrafish zDHHC13 regulates embryonic differentiation through interaction with Smad6, zDHHC15b is implicated in the differentiation of dopamine (DA) neurons, and the anchor protein domain of zDHHC17 influences neuronal axon growth by regulating the formation of the TrkA tubulin complex (17, 19, 20). Only zDHHC16 promotes telencephalic neural stem cell proliferation by modulating the FGF/ERK signaling pathway through its PAT activity (18). Research on the immune regulatory roles of zDHHCs in teleosts is further limited. Only zDHHC1, identified in grass carp (*Ctenopharyngodon idella*) and Chinese perch (*Siniperca chuatsi*), has been reported to possess antiviral functions (21, 22).

In the current study, we systematically screened the large yellow croaker genome for members of the zDHHC family, identifying 22 zDHHC proteins (*Lc*zDHHC1–9, 11–18, 20–24). Subsequently, we cloned all 22 zDHHCs and conducted analyses on their evolutionary relationships, protein structures, and importantly, their potential association with *Pseudomonas plecoglossicida* (*P. plecoglossicida*) infection, a significant pathogenic bacterium affecting large yellow croaker (23). Among them, *LczDHHC23* exhibited the highest upregulation in *P. plecoglossicida* infected large yellow croaker macrophage/monocyte cells (MO/MΦ). However, the immune regulatory functions of zDHHC23 have not been previously explored, even in mammals. In mammals, zDHHC23 primarily palmitoylates molecules implicated in mTOR signaling and tumorigenesis (24, 25). Therefore, investigating the mechanisms underlying the notable upregulation of *LczDHHC23* expression post-infection holds significance. By silencing *Lc*zDHHC23 expression, we observed an upregulation of pro-inflammatory cytokines and a downregulation of anti-inflammatory cytokines during *P. plecoglossicida* infection, indicating the anti-inflammatory role of *Lc*zDHHC23. Further investigation revealed that *Lc*zDHHC23 predominantly promotes M2-type macrophage polarization while inhibiting M1-type polarization, as also evidenced by enhanced phagocytic activity in *Lc*zDHHC23-knockdown MO/MΦ. Additionally, *Lc*zDHHC23 was found to facilitate the necrosis of *P. plecoglossicida*-infected MO/MΦ, as evidenced by delayed and reduced phosphorylation of necrosis markers receptor−interacting serine/threonine kinase (RIP)1, RIP3, and mixed lineage kinase domain-like (MLKL) upon compromised *Lc*zDHHC23 expression. To our knowledge, our investigation represents the first study to elucidate the role of zDHHC23 in immune cells during infection, potentially laying the groundwork for a deeper understanding of zDHHC23’s function in modulating anti-pathogen immune responses.

## 2 Materials and methods

### 2.1 Sampling and challenging

Healthy large yellow croakers (100–120 g) were purchased from Xiangshan county farm (NingBo, China). All fish were temporarily kept in a recirculating seawater system maintained at 18–20°C for at least two weeks. All animal experiments were approved by the Institutional Animal Care and Use Committee of Ningbo University and conducted in accordance with the Guide for the Care and Use of Laboratory Animals issued by the National Institutes of Health. *P. plecoglossicida* strain (NZBD9) was cultivated at 18°C in Luria–Bertani (LB) broth with shaking and collected until reaching the logarithmic growth stage. The bacteria were washed with sterile phosphate-buffered solution (PBS), and then diluted to a final 5 ×10^4^ colony forming units (CFU)/100g fish in 100 μL PBS for the *in vivo* challenge test. The large yellow croakers were intraperitoneally injected with *P. plecoglossicida*, while the control group received an equivalent volume of PBS. The tissue samples in each group (gill, head kidney, intestine, liver, and spleen) were collected at 0, 6, 12, 24, 48 and 72 hours after infection (hpi), and promptly snap-frozen in liquid nitrogen and stored at -80°C for subsequent Real-time quantitative polymerase chain reaction (RT-qPCR) analysis. The primers used are listed in **Supplementary Table 1**.

### 2.2 Cloning of the *Lc*zDHHC genes and bioinformatic analysis

The 22 *Lc*zDHHC genes were cloned via reverse transcription PCR (RT-PCR), using sequences obtained from the NCBI database. Subsequently, the PCR products were sequenced for validation. The primers used are listed in **Supplementary Table 1**. Phylogenetic analysis of the whole family was conducted using the neighbor-joining method, supported by 1000 bootstrap repetitions in MEGA 7.0 software. The phylogenetic tree was modified using iTOL programs. Transmembrane (TM) domain analysis of *Lc*zDHHC23 was predicted by the TMHMM Server v. 2.0 program (http://www.cbs.dtu.dk/services/TMHMM-2.0/). Multiple sequence alignment was generated using ClustalW (http://clustalw.ddbj.nig.ac.jp/). The domain information of *Lc*zDHHC23 was predicted using SMART (http://smart.embl-heidelberg.de/), and the three-dimensional structures of all *Lc*zDHHCs were predicted using SWISS-MODEL (https://swissmodel.expasy.org/), followed by visualization of the PDB files using PyMOL software version 3.1. Sequences used in this study are listed in **Supplementary Table 2**.

### 2.3 Large yellow croaker head-kidney-derived MO/MΦ isolation and *P.plecoglossicida* stimulation

Large yellow croaker head-kidney-derived MO/MΦ were isolated as previously described (26, 27). Leukocyte-enriched fractions were obtained by applying dissociated head-kidney to a Ficoll density gradient (1.077 g/mL; #17144002 GE Healthcare, Chicago, IL, USA). The cells were then seeded in 6 well plates at a density of 2×10^7^/mL and cultured overnight at 26°C under 5% CO2. After washing away the nonadherent cells, attached cells were incubated with complete DMEM medium (10% fetal bovine serum, 100 U/mL penicillin, and 100μg/mL streptomycin) and cultured under the same conditions. Live *P. plecoglossicida* diluted in PBS at a multiplicity of infection (MOI) of 2 or PBS alone were added into cell culture medium. Cells were collected at 4, 8, 12, and 24 hpi for RNA extraction, and RT-qPCR was conducted as described. The primers used are listed in **Supplementary Table 1**.

### 2.4 RNA extraction and RT-qPCR

Total RNA of both large yellow croaker tissues and MO/MΦ were isolated using RNAiso (#9108/9109, TaKaRa, Dalian, China), treated with DNase I (#2270A, TaKaRa), and reversed transcribed into first-strand cDNA using AMV reverse transcriptase (#2621, TaKaRa) according to manufacturer protocol. RT-qPCR was conducted on an ABI StepOne real-time PCR system (Applied Biosystems, Foster City, CA, USA) using SYBR premix Ex Taq II (#RR82WR, TaKaRa). The thermal cycling conditions were as follows: initial denaturation at 95°C for 10 s, followed by 40 cycles of amplification (95°C for 50 s and 60°C for 20 s), and final melting curve analysis (95°C for 60 s, 55°C for 30 s, and 95°C for 30 s). Relative gene expression was calculated using the 2^−ΔΔCT^ method and the data were normalized against *Lc*18S rRNA. The primers used are listed in **Supplementary Table 1**. Each PCR trial was performed in triplicate and repeated at least three times.

### 2.5 Constructing of *Lc*zDHHC23 eukaryotic expression plasmids

The full-length open reading frame (ORF) of *Lc*zDHHC23 was amplified by PCR using PrimeSTAR GXL DNA polymerase (#R050A, Takara). The PCR product was ligated with pcDNA-HA vector to generate the HA-tagged *Lc*zDHHC23 plasmid using the *pEASY* ^®^-Basic Seamless Cloning and Assembly Kit (#CU201–02, TransGen Biotech). The plasmid was subsequently transformed into competent *Escherichia coli* (*E. coli*) cells. The bacterial solution was spread onto LB nutrient agar plates, and single colonies were selected from overnight cultures for sequencing.

### 2.6 Subcellular localization of *Lc*zDHHC23

Hela cells were seeded at a density of 1×10^5^/mL and transfected with HA-tagged *Lc*zDHHC23 plasmids. After 36 h of transfection, the cells were fixed with 4% paraformaldehyde in PBS (pH=7.4) and permeabilized with 0.5% saponin. After blocking, the cells were incubated with appropriate primary and secondary antibodies. Confocal images were obtained using Zeiss LSM 880 confocal microscope (Carl Zeiss AG, Oberkochen, Germany) and analyzed using the ZEN Blue software.

### 2.7 RNA interference

*Lc*zDHHC23-specific small interfering RNA (RiboBio, Guangzhou, China) was transfected into large yellow croaker MO/MΦ using Lipofectamine™ RNAiMAX Transfection Reagent (#13778150, Invitrogen/Life Technologies, Carlsbad, CA, USA) according to the previous studies for 24 and 48 h to evaluate the knock down efficiency (21, 28–30). The scrambled siRNA was used as the control. To evaluate the role of *Lc*zDHHC23 on MO/MΦ function following *P. plecoglossicida* stimulation, the isolated MO/MФ were transfected with 30 pmol *Lc*zDHHC23 siRNA, or scrambled siRNA for 24h before infected with *P.plecoglossicida*.

### 2.8 *In vitro* bacterial-killing assay

Isolated MO/MΦ were transfected with *Lc*zDHHC23 siRNA, or corresponding controls, for 24 h before being infected with live *P. plecoglossicida* at an MOI of 4 (30, 31). The phagocytosis of bacteria was proceeded for 30 minutes at 26°C under 5% CO_2_. The remaining bacteria attached to cell surface were killed using gentamicin (50 μg/mL), and then washed with sterile PBS. Each set of interfered MO/MФ were divided into two groups. One group (the uptake group) was lysed immediately with 1% Triton X-100 solution and plated onto solid LB agar medium to assess bacterial uptake. The other group (the kill group) was incubated for an additional 1.5 h before being lysed and plated on LB agar medium. After incubation at 28°C for 24 h, the CFUs of the plates were calculated. Bacterial survival was determined by dividing the CFUs in the kill group by those in the uptake group. Three independent experiments were performed.

### 2.9 MO/MФ polarization assay

To investigate the impact of *Lc*zDHHC23 on MO/MФ polarization, LPS-induced M1-type and cAMP-induced M2-type MO/MФ were prepared according to previous reported (32). MO/MФ cells were transfected with *Lc*zDHHC23 siRNA, or corresponding controls for 24 h before treatment with LPS (50 μg/mL; #L4391, Sigma Aldrich) or a cAMP analog (dibutyryl cAMP; 0.5 mg/mL, #28745-M, Sigma-Aldrich) for 18 h. Expression levels of the pro-inflammatory cytokines (*IL-1β* and *IL-6*) and the anti-inflammatory cytokines (*IL-10* and *TGF-β*) were determined. Moreover, the markers of M1-type MO/MФ (*C-X-C motif chemokine ligand 9* (*CXCL9*) and *induced nitric oxide synthase* (*iNOS*)) and the markers of M2-type MO/MФ (*secreted phosphoprotein 1* (*SPP1*) and *arginase*) were also evaluated (33). In addition, the iNOS (M1-type) and arginase activities (M2-type) were assessed. iNOS activity was measured using a nitric oxide synthase assay kit (fluorescence probe method; #S0024, Beyotime, Shanghai, China) following the manufacturer’s instructions. The relative iNOS activity of each group was expressed as fold change relative to the value of the control group. Arginase activity was measured using an arginase activity assay kit (#MAK112, Sigma-Aldrich) following the manufacturer’s protocol. Absorbance was read at 430 nm, and arginase activity (U/L) was calculated according to comparison with urea-standard data.

### 2.10 Necroptosis assay

Large yellow croaker MO/MΦ were transfected with *Lc*zDHHC23 siRNA, or corresponding controls, for 24 h prior to infection with *P. plecoglossicida* at an MOI of 10 (30, 31). Cell samples were collected at 1, 2, 4 and 8 hpi. The collected cells were washed and then labeled with Annexin V-FITC and propidium iodide (PI) for 15 minutes using a FITC Annexin V apoptosis detection kit I (#556547, BD Pharmingen, San Diego, CA, USA). Apoptosis was evaluated by flow cytometry using the MACSQuant Analyzer 10 (Miltenyi Biotec) within 15 min of staining, and data were analyzed using MACSQuant analysis software (Miltenyi Biotec). Three independent experiments were performed.

### 2.11 Western blotting

Large yellow croaker MO/MΦ cells were seeded in 6 well plates at a density of 2×10^7^/mL the day before the experiment. Cells were washed twice with PBS and infected with *P.plecoglossicida* at an MOI of 10. Subsequently, cells were collected at 0, 1, 2, 4 and 8 hpi and lyzed by lysis buffer (20 mM Tris-HCl [pH=8.0], 2 mM EDTA, 120 mM NaCl, 1% NP-40) containing phosphatase inhibitor (#A32963, Thermo fisher). The soluble protein concentration was measured using the Bradford method. Proteins were separated on 10% or 15% SDS-PAGE, transferred to polyvinylidene difluoride (PVDF) membranes (#IPVH00010, EMD Millipore). The membranes were blocked with 5% non-fat milk for 1 h at room temperature, then incubated overnight at 4°C with apoptosis/necroptosis antibodies (#92570, Cell Signaling Technology), followed by incubation with appropriate HRP-conjugated secondary antibodies for 1 h at room temperature. The blots were subsequently visualized using a chemiluminescent detection system with ECL western blotting detection reagents (#32106, Thermo Fisher Scientific).

### 2.12 Statistical analysis

All data are presented as the means ± SEM. Statistical analysis was performed using one-way analysis of variance (ANOVA) with the Prism 8.02 software (GraphPad Software, San Diego, CA, USA). The p values *p < 0.05 were considered statistically significant.

## 3 Results

### 3.1 Cloning and bioinformatic analysis of the *Lc*zDHHC members

**Figure 1:**
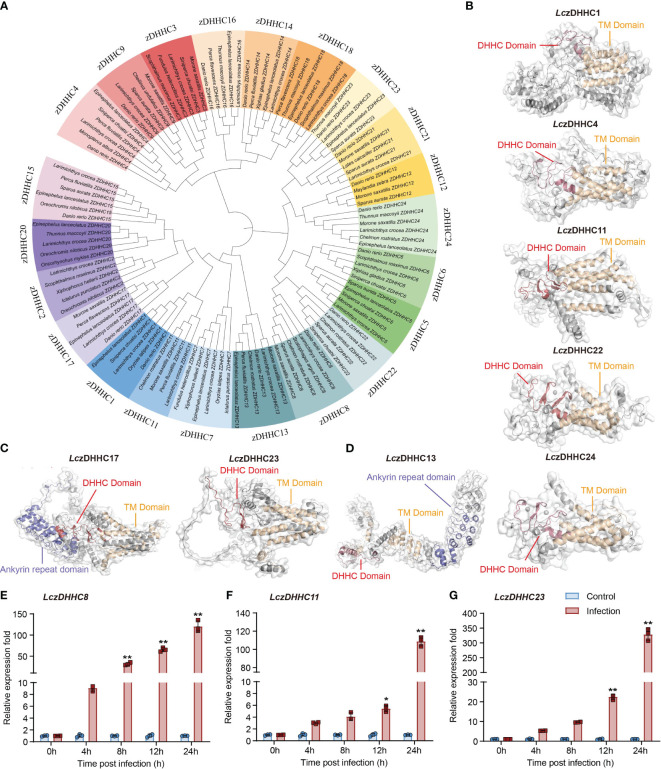
Bioinformatic analysis and expression of *Lc*zDHHC family in response to *P. plecoglossicida* infection in head kidney-derived MO/MΦ. **(A)** Phylogenetic tree showing the divergence of zDHHC proteins among teleost species, generated using the neighbor-joining method in MEGA 7.0 with 1000 bootstrap replications and modified using iTOL. **(B–D)** Predicted tertiary structures of *Lc*zDHHC family proteins with five **(B)**, six **(C)**, and seven **(D)** transmembrane (TM) domains. Structures were modeled using SWISS-MODEL and visualized using PyMOL software version 3.1. *Lc*zDHHC 1, 4, 11, 22, and 24 have five TM domains **(B)**, *Lc*zDHHC17 and *Lc*zDHHC 23 have six TM domains **(C)**, and *Lc*zDHHC13 has seven TM domains **(D)**. Additionally, *Lc*zDHHC13 and 17 feature an ankyrin repeat domain. TM domains are depicted in yellow, DHHC domains in red, and ankyrin repeat domains in deep blue. **(E–G)** Quantitative analysis of significantly upregulated expression of *LczDHHCs* in response to *P. plecoglossicida* infection, including *LczDHHC8* **(E)**, *LczDHHC11* **(F)**, and *LczDHHC23* **(G)**. MO/MΦ from large yellow croakers were infected with *P. plecoglossicida* at an MOI of 2, with PBS-treated cells as controls. Samples were collected at 0, 4, 8, 12, and 24 hpi. Expression levels of each *LczDHHC* mRNA were normalized to *Lc18S rRNA* and then to the 0 h PBS control using the 2^-ΔΔCT^ method. Data represent the means ± SEM of three replicates. *p < 0.05 and **p < 0.01.

Twenty-two *Lc*zDHHCs have been cloned from the large yellow croaker (*Lc*zDHHC1–9, 11–18, 20–24). Unlike observed in mammals, the zDHHC family in the large yellow croaker does not include zDHHC19 and zDHHC25 (1). Phylogenetic analysis revealed three major clades grouping the 22 subfamilies, with each of these 22 *Lc*zDHHCs forming clusters with their respective orthologues from other teleost species (**Figure 1A**). The tertiary structures of the *Lc*zDHHCs were predicted using SWISS-MODEL, revealing a highly conserved DHHC domain and 4 to 7 transmembrane (TM) domains in all members (**Figures 1B–D**; **Supplementary Figure 1**). Additionally, *Lc*zDHHC6 contains an SH3 domain, while *Lc*zDHHC13 and *Lc*zDHHC17 feature an ankyrin repeat domain. The TM helices of these *Lc*zDHHCs form pocket-like structures, and the DHHC domain contains 2 or more β-hairpin structures, except for *Lc*zDHHC13. This arrangement is reminiscent of the crystal structure of zDHHC proteins reported in humans and zebrafish (34). Typically, the DHHC domain of most *Lc*zDHHC proteins (excluding *Lc*zDHHC13, 17, and 23) is situated between the second and third TM domains (**Figures 1B–D**; **Supplementary Figure 1**).

### 3.2 *Lc*zDHHC23 exhibits the most significant response to *P. plecoglossicida* infection among all zDHHCs

We screened the 22 *Lc*zDHHCs for changes in expression during *P. plecoglossicida* infection in large yellow croaker head kidney-derived MO/MΦ. Among them, *Lc*zDHHC8, *Lc*zDHHC11, and *Lc*zDHHC23 showed significant induction, with *Lc*zDHHC23 exhibiting the highest induction, reaching over 300-fold at 24 hpi (**Figures 1E–G**). The expression changes of other *Lc*zDHHCs were minor, with induction or suppression within 20-fold (**Supplementary Figures 2**, **3**). Consequently, we focused on studying the role of *Lc*zDHHC23 in regulating the immune response induced by *P. plecoglossicida* infection in the subsequent investigation.

**Figure 2:**
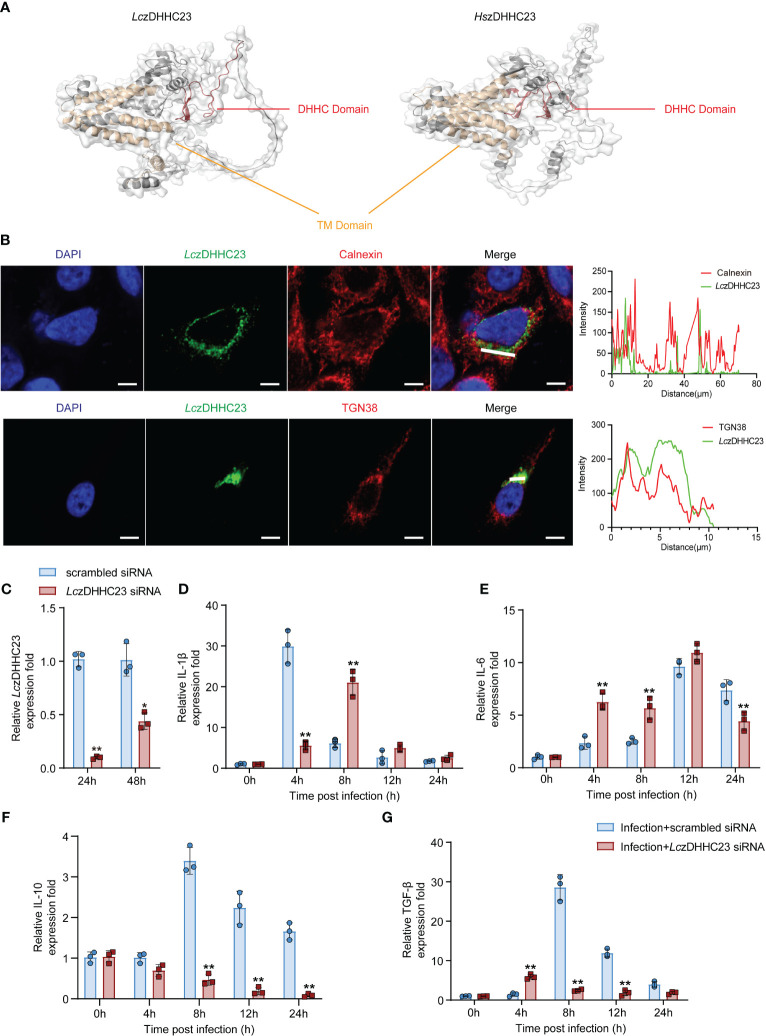
Molecular characterization of *Lc*zDHHC23 and its impact on cytokine expression of *P. plecoglossicida-*infected MO/MΦ. **(A)** Comparison of the tertiary structures of *Lc*zDHHC23 and *Homo sapiens* zDHHC23 (*Hs*zDHHC23). The tertiary structures were modeled using SWISS-MODEL and visualized using PyMOL software version 3.1. TM domains are represented in yellow, and DHHC domain is depicted in red. **(B)** Immunofluorescence images showing the subcellular localization of *Lc*zDHHC23 with the endoplasmic reticulum marker Calnexin and Golgi apparatus marker TGN38. HeLa cells were seeded at a density of 1 × 10^5^/mL and transfected with HA-tagged *Lc*zDHHC23 plasmid. After 36 h of transfection, the cells were fixed and examined using a confocal microscope. The white line indicates the color pickup line, with the fluorescence intensity curve shown in green and red on the right side, analyzed with ZEN software. Scale bars, 5 μm. Results are representative of three independent experiments. **(C–G)** Impact of *Lc*zDHHC23 on the cytokine expression in *P. plecoglossicida* infected MO/MΦ. **(C)** RT-qPCR assesses the interference efficiency of *Lc*zDHHC23 siRNA. Transcript levels of *LczDHHC23* were normalized against *18S rRNA* and then to the 24 h scrambled siRNA control group using the 2^-ΔΔCT^ method. *p < 0.05 and **p < 0.01. **(D–G)** RT-qPCR analysis evaluates the effects of *Lc*zDHHC23 interference on cytokine mRNA expression in *P. plecoglossicida-*infected MO/MΦ. Large yellow croaker MO/MΦ were transfected with *Lc*zDHHC23 siRNA for 24 h before *P. plecoglossicida* infection at an MOI of 2. Samples were collected at 0, 4, 8, 12, and 24 hpi. Expressions levels of *IL-1β*, *IL-6*, *IL-10*, and *TGF-β* were detected using RT-qPCR. mRNA expression was normalized to that of *18S rRNA* and then to the respective 0 h control using the 2^-ΔΔCT^ method. Data represent the means ± SEM of three replicates. *p < 0.05 and **p < 0.01.

The *Lc*zDHHC23 sequence spans 2355 nucleotides (nt), with an open reading frame (ORF) of 1178 base pairs (bp), encoding a protein of 392 amino acids (aa). A phylogenetic tree was constructed based on the amino acid sequences of zDHHC23 proteins from teleosts and other species. The analysis revealed that teleost zDHHC23s formed a distinct cluster, with *Lc*zDHHC23 showing the closest relationship to that of *Nibea albiflora* (**Supplementary Figure 4A**). Multiple sequence alignment demonstrated a high degree of conservation among zDHHC23 orthologs, comprising six transmembrane (TM) domains and a highly conserved DHHC domain (**Supplementary Figure 4B**). We further compared the tertiary structure of *Lc*zDHHC23 with that of human orthologue (*Hs*zDHHC23), and found that they both possess six TM helices, forming a pocket-like structure similar to that of most members of the zDHHC family. Additionally, they feature a highly conserved DHHC domain positioned between the fourth and fifth TM domains, contrasting with most zDHHC family proteins where it situated between the second and third TM domains. As depicted in **Figure 2A**, the DHHC domain of *Lc*zDHHC23 contains two β-hairpin structures, whereas the DHHC domain of *Hs*zDHHC23 has more. This suggests that the DHHC domain of *Hs*zDHHC23 has a more complex structure compared to that of the *Lc*zDHHC23.

### 3.3 Subcellular localization and tissue distribution pattern of *Lc*zDHHC23

We further evaluated the molecular characteristics of *Lc*zDHHC23. Previous studies have indicated that members of the zDHHC family primarily localize to the endoplasmic reticulum, the Golgi apparatus, or both (35). Our subcellular localization analysis via immunofluorescence revealed that *Lc*zDHHC23 predominantly co-localizes with the Golgi apparatus marker TGN38, rather than the endoplasmic reticulum marker Calnexin (**Figure 2B**), indicating a difference from observations in mammals (36). The tissue expression pattern of *Lc*zDHHC23 in major immune tissues (intestine, head kidney, liver, gill, and spleen) of healthy large yellow croaker revealed that the spleen exhibited the highest levels of *Lc*zDHHC23 transcripts, followed by the liver and head kidney (**Supplementary Figure 5A**). Following *P. plecoglossicida* injection, the expression of *Lc*zDHHC23 significantly increased in all examined tissues except for the liver, which showed a decrease at 12 hpi. The highest expression levels of *Lc*zDHHC23 in the head kidney and gill were observed at 12 hpi, followed by a decrease. In the intestine, *Lc*zDHHC23 expression initially decreased significantly and then sharply increased. *Lc*zDHHC23 levels in the spleen increased at 12 hours and remained stable until 72 hpi (**Supplementary Figures 5B–F**).

### 3.4 *Lc*zDHHC23 plays an anti-inflammatory role in MO/MΦ during *P. plecoglossicida* infection

To assess the impact of *Lc*zDHHC23 on immune responses following *P. plecoglossicida* infection, we initially synthesized *Lc*zDHHC23 siRNA (5’-CACCACTGCATCTGGATAA-3’) to downregulate its expression in large yellow croaker MO/MΦ. The expression of *Lc*zDHHC23 was notably reduced after 24 and 48 hours of interference compared to the control group, reaching 9.8% and 43.8%, respectively (**Figure 2C**). After confirming the efficacy of *Lc*zDHHC23 siRNA knockdown, we investigated the impact of *Lc*zDHHC23 expression interference on the expression of pro-inflammatory (*IL-1β* and *IL-6*) and anti-inflammatory (*IL-10* and *TGF-β*) cytokines following *P. plecoglossicida* infection. The results revealed that inhibiting *Lc*zDHHC23 significantly upregulated the expression of pro-inflammatory cytokines (*IL-1β* and *IL-6*; **Figures 2D, E**) and downregulated the expression of anti-inflammatory cytokines (*IL-10* and *TGF-β*; **Figures 2F, G**), compared to the control group. These findings suggest that *Lc*zDHHC23 may indeed play an anti-inflammatory role in MO/MΦ during *P. plecoglossicida* infection in large yellow croaker.

### 3.5 *Lc*zDHHC23 promotes M2-type polarization of large yellow croaker MO/MΦ

**Figure 3:**
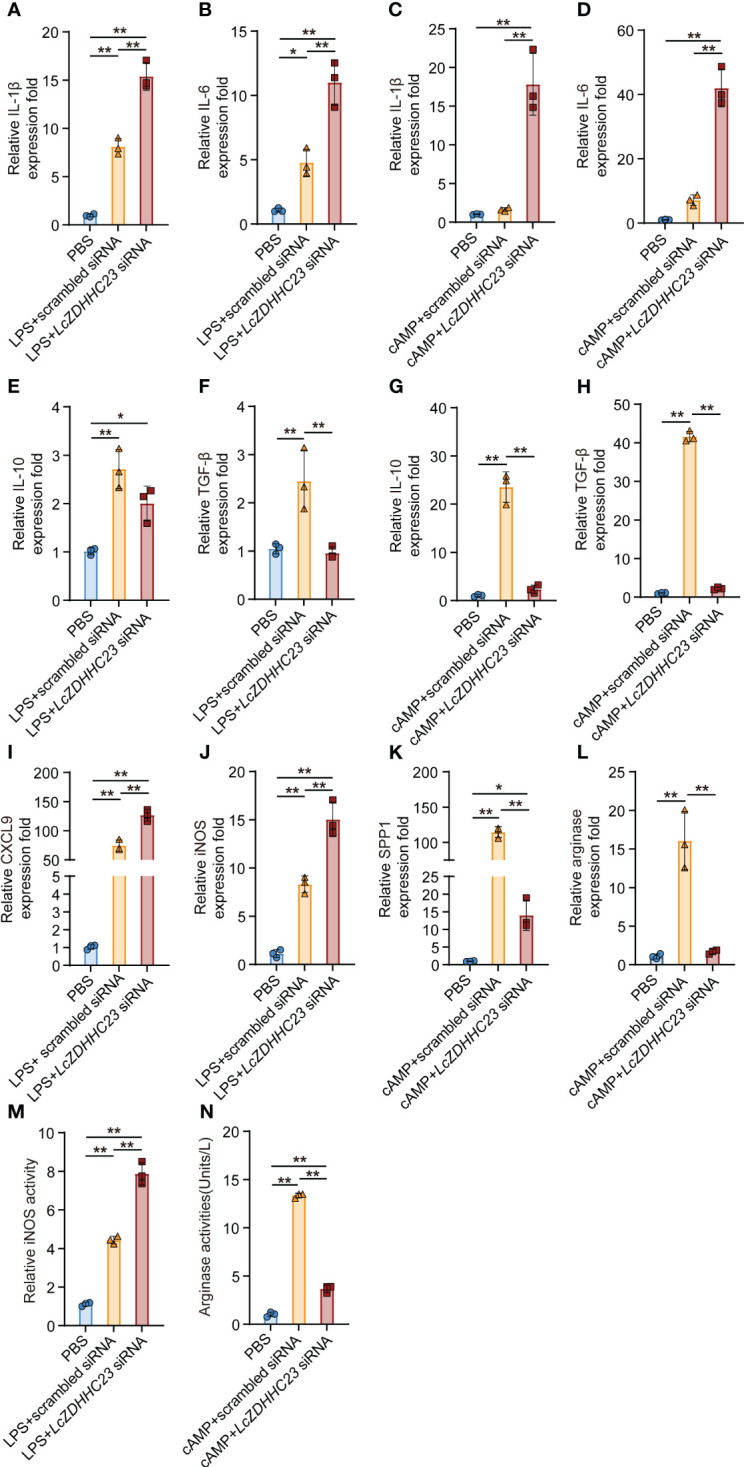
Effect of *Lc*zDHHC23 deficiency on the polarization of large yellow croaker MO/MФ. Large yellow croaker MO/MΦ were transfected with *Lc*zDHHC23 siRNA for 24 h before stimulation with LPS or cAMP for 18 h. **(A, B, E, F)** Knockdown of *Lc*zDHHC23 promotes M1-type polarization of MO/MΦ. Silencing *Lc*zDHHC23 significantly induced the expression of pro-inflammatory cytokines *IL-1β* **(A)** and *IL-6* **(B)**, while decreased the expression of anti-inflammatory cytokines *IL-10* **(E)** and *TGF-β* **(F)** following LPS stimulation. **(I, J, M)** Expression of M1-type MO/MФ markers *CXCL9* **(E)**, *iNOS* **(F)**, as well as the iNOS activity **(G)** were increased with *Lc*zDHHC23 knockdown. **(C, D, G, H)** *Lc*zDHHC23 knockdown inhibits the M2-type polarization of large yellow croaker MO/MΦ. Knockdown of *Lc*zDHHC23 significantly increased the expression of pro-inflammatory cytokines *IL-1β* **(C)** and *IL-6* **(D)**, while decreasing the expression of anti-inflammatory cytokines *IL-10* **(G)** and *TGF-β* **(H)** following cAMP stimulation. **(K, L, N)** Expression of M2-type MO/MФ markers *SPP1* **(K)**, *arginase* **(L)**, as well as the arginase activity **(N)** were also decreased with *Lc*zDHHC23 inhibition. Data represent the means ± SEM of three replicates. *p < 0.05 and **p < 0.01.

We further evaluated whether *Lc*zDHHC23 exert its anti-inflammatory roles by affecting the polarization of large yellow croaker MO/MΦ. Fish MO/MФ can differentiate into two main types: the M1-type, characterized by the expression of pro-inflammatory cytokines and the production of reactive oxygen species (ROS) and NO, and the M2-type, characterized by the expression of anti-inflammatory cytokines and increased arginase activity (33, 37). We induced M1-type and M2-type polarization of isolated large yellow croaker MO/MΦ using lipopolysaccharide (LPS) and cyclic adenosine monophosphate (cAMP), respectively, as previously reported (32). As shown in **Figure 3**, knockdown of *Lc*zDHHC23 further promoted the induced expression of pro-inflammatory cytokines (*IL-1β* and *IL-6*) and M1-type markers (*CXCL9 and iNOS*), as well as the iNOS activity in M1-type MO/MΦ (**Figures 3A, B, I, J, M**). Conversely, *Lc*zDHHC23 silencing further decreased the already low expression of anti-inflammatory cytokines (*IL-10* and *TGF-β*) in M1-type MO/MΦ, indicating an inhibiting role of *Lc*zDHHC23 on the M1-type function (**Figures 3E, F**). Moreover, *Lc*zDHHC23 silencing significantly upregulated the expression of *IL-1β* and *IL-6* in cAMP-induced M2-type MO/MΦ, while downregulating the expression of *IL-10, TGF-β*, as well as the M2 marker *SSP1*, and the arginase activity (**Figures 3C, D, G, H, K, L, N**). These findings suggest that *Lc*zDHHC23 may promote M2-type MO/MΦ polarization to exert its anti-inflammatory function. In support of this, we observed a significant augmentation in bacterial killing activity in *Lc*zDHHC23-silenced MO/MΦ. Specifically, the bacterial killing activity decreased from 22.84% to 6.07% in *Lc*zDHHC23-deficient cells compared to control cells (**Supplementary Figure 6**).

### 3.6 *Lc*zDHHC23 augments the necroptosis of *P. plecoglossicida* infected MO/MΦ

**Figure 4:**
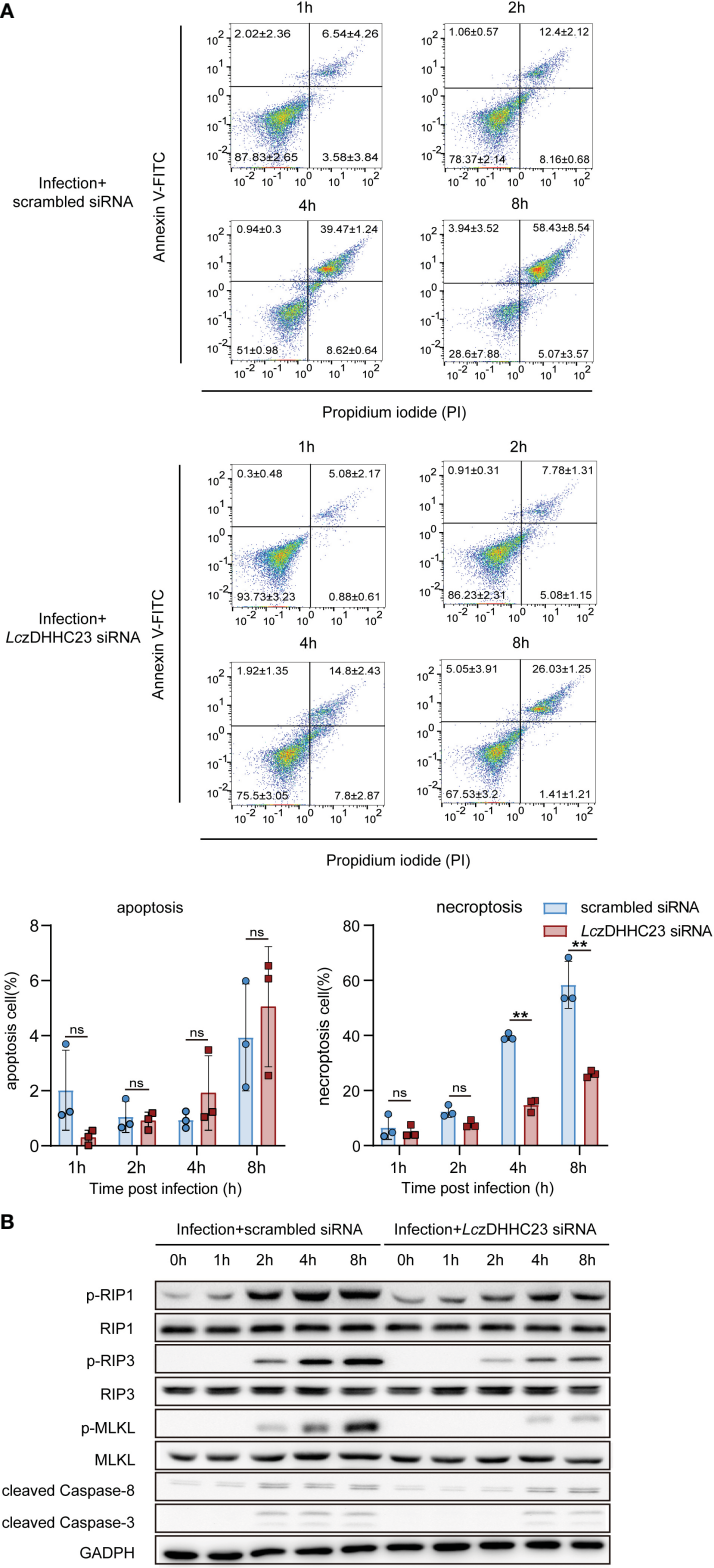
Impact of *Lc*zDHHC23 deficiency on *P. plecoglossicida-*induced necroptosis of large yellow croaker MO/MФ. **(A)** MO/MΦ were transfected with *Lc*zDHHC23 siRNA for 24 h prior to *P. plecoglossicida* infection at an MOI of 10. After 1, 2, 4 and 8 h of infection, cells were harvested and stained with Annexin V-FITC and PI for flow cytometry analysis. Live cells, are negative for both PI and Annexin V-FITC (lower left quadrant); dead cells are positive for PI but negative for Annexin V-FITC (lower right quadrant); necroptotic cells are positive for both PI and Annexin V-FITC (upper right quadrant), while early apoptotic cells are positive for Annexin V-FITC but negative for PI (upper left quadrant). Histograms represent the percentage of apoptosis and necroptosis. Data represent the means ± SEM of three replicates. **p < 0.01, ns represent not significant. **(B)** Cells collected from the above experiment were also collected and lysed for Western blot analysis to evaluate the expression of necroptosis-related proteins (RIP1, RIP3, MLKL, p-RIP1, p-RIP3, and p-MLKL) and apoptosis-related proteins (cleaved Caspase-3 and cleaved Caspase-8). GAPDH was used as an internal control.

Given *Lc*zDHHC23’s role in promoting M2-type polarization and anti-inflammatory responses in large yellow croaker MO/MΦ, and considering the impact of inflammatory responses on cell states during pathogen infection (38), we further assessed the involvement of *Lc*zDHHC23 in the apoptosis/necroptosis of *P. plecoglossicida*-infected large yellow croaker MO/MΦ. The findings indicate that *P. plecoglossicida* infection predominantly triggers necroptosis in MO/MΦ, with the proportion of necroptotic cells gradually increasing during infection, reaching 58.43 ± 8.54% at 8 hpi. However, upon silencing of *Lc*zDHHC23, the induction of MO/MΦ necroptosis by *P. plecoglossicida* infection was significantly reduced, with only 26.03 ± 1.25% of cells undergoing necroptosis at 8 hpi (**Figure 4A**). We further validated the impact of *Lc*zDHHC23 on *P. plecoglossicida* infection-induced MO/MΦ necroptosis by examining necroptosis markers, including phosphorylated RIP3 (p-RIP3), RIP1 (p-RIP1), and MLKL (p-MLKL), as well as assessing Caspase-3/8 activity inhibition (39). Results demonstrated a significant increase in the phosphorylation levels of these proteins at 2 hpi, with further elevation observed as the infection progressed. Concurrently, cleaved Caspase-3 and Caspase-8 remained at very low levels, with minimal observation during the late stage of infection when signs of apoptosis were also noted (**Figures 4A, B**). Upon silencing of *Lc*zDHHC23, the heightened phosphorylation of RIP1, RIP3, and MLKL induced by *P. plecoglossicida* infection was mitigated or delayed (**Figure 4B**), indicating the involvement of *Lc*zDHHC23 in promoting immune cell necroptosis during pathogen infection.

## 4 Discussion

Previous studies have underestimated the non-PAT immunomodulatory functions of zDHHC family proteins, but primarily focused on the palmitoylation modification of immune-related molecules mediated by the zDHHC members (11, 15, 16). Also, research on the immune regulatory roles of zDHHCs in teleosts remains limited, with only zDHHC1 molecules investigated for their antiviral functions (21, 22). Herein, we identified 22 zDHHC proteins in the large yellow croaker genome, excluding zDHHC10 and 19, and analyzed their evolutionary relationships. Interestingly, we observed the absence of the zDHHC19 gene in teleosts, contrasting with its presence in mammals (1). Among the identified zDHHC proteins, *Lc*zDHHC23 stood out due to its significant upregulation following *P. plecoglloscida* infection in MO/MΦ, prompting further investigation into its potential immune regulatory role. Our choice to focus on *Lc*zDHHC23 was also influenced by previous studies implicating zDHHC23 in immune-related processes, particularly its involvement in mTOR signaling and tumorigenesis (24, 25). Thus, we aimed to elucidate the specific immune functions of *Lc*zDHHC23 in the context of pathogen infection induced immune responses in teleosts.

Our bioinformatics analyses provided insights into the functional domains and evolutionary patterns of *Lc*zDHHC23. Phylogenetic analyses revealed its clustering with zDHHC23 from teleosts, particularly *Nibea albiflora*, suggesting evolutionary conservation. Furthermore, structural predictions indicated similarities between *Lc*zDHHC23 and *Hs*zDHHC23 proteins, particularly in their DHHC domains and transmembrane regions. Despite these similarities, we noted differences in the subcellular localization of *Lc*zDHHC23, predominantly within the Golgi apparatus in contrast to mammalian studies (36).

In examining the immune function of *Lc*zDHHC23, we observed its widespread expression in immune-related tissues of healthy large yellow croaker, with significant upregulation following *P. plecoglloscida* infection, except in the liver. The expression pattern of *Lc*zDHHC23 initially increased, followed by a decrease, in both the liver and gills after *P. plecoglossicida* infection. This suggests that the immune system might boost immune cell activity and inflammatory signaling pathways by upregulating the expression of the *zDHHC23* gene to combat pathogen invasion, while its gradual decrease in expression aims to alleviate excessive inflammation and immune-related damage (40). These findings underscore the involvement of *Lc*zDHHC23 in the immune response against bacterial infection (41, 42). Silencing *Lc*zDHHC23 expression in MO/MΦ resulted in heightened pro-inflammatory cytokine expression and reduced anti-inflammatory cytokine levels during *P. plecoglossicida* infection, indicating its anti-inflammatory role. Meanwhile, we observed a significant increase in the expression of *TGF-β* at 4 h, whereas the expression of *IL-1β* and *IL-6* significantly decreased at 4 and 24 h, respectively. Typically, MO/MΦ release inflammatory cytokines rapidly upon pathogen infection. However, to mitigate excessive inflammation and immune damage, the expression of inhibitory inflammatory cytokines may gradually rise (43). This result could be attributed to the regulatory role of zDHHC23, a non-PAT, in the cytokine signaling pathway. Currently, there is insufficient literature on the non-PAT functions of the zDHHC family and their involvement in cytokine signaling pathways. Further research is warranted to elucidate specific targets and underlying mechanisms.

Moreover, our study revealed the impact of *Lc*zDHHC23 deficiency on MO/MΦ polarization, shifting the balance towards the pro-inflammatory M1 phenotype. These findings further support the anti-inflammatory role of *Lc*zDHHC23 in MO/MΦ and its role in promoting M2-typ MO/MΦ polarization (32, 44). Further, silencing *Lc*zDHHC23 reduced *P. plecoglloscida* infection induced necroptosis of MO/MΦ, which was also supported by decreased phosphorylation levels of necroptosis markers, suggesting a protective role against cell death with *Lc*zDHHC23 deficiency (45, 46). Meanwhile, we also observed a slight decrease in the expression of cleaved Caspase-3 and 8 in the *Lc*zDHHC23 knockout group 2 hpi (**Figure 4B**). However, no further significant differences were observed with prolonged infection. Furthermore, upon calculating the relative grey intensity of the Cleaved Caspase-3 band to that of GAPDH (data not shown), we found that the minor decrease of the 2 hpi band were not statistically significant. We thought that the weak cleavage of Caspase-3 may not be significant enough to indicate a notable effect. Additionally, our statistical analysis of flow cytometry data representing apoptosis revealed no significant difference between the *Lc*zDHHC23 knockdown and control groups (**Figure 4A**). Therefore, we thought the influence of *Lc*zDHHC23 on cell death induced by infection primarily pertains to necroptosis. Given the emerging role of necroptosis in host defense against pathogens, elucidating the precise mechanisms underlying *Lc*zDHHC23-mediated regulation of necroptosis could provide valuable insights into host-pathogen interactions in teleosts.

In summary, our study elucidates the immune regulatory functions of *Lc*zDHHC23 in large yellow croaker, highlighting its anti-inflammatory properties in MO/MΦ. By modulating cytokine expression, polarization, and necroptosis, *Lc*zDHHC23 plays a crucial anti-inflammatory role in the immune response against bacterial infection. However, further investigations are warranted to unravel the precise mechanisms underlying *Lc*zDHHC23-mediated immune regulation and its potential as a therapeutic target for immune modulation in teleosts.

## Data availability statement

The datasets presented in this study can be found in online repositories. The names of the repository/repositories and accession number(s) can be found in the article/**Supplementary material**.

## Ethics statement

The animal study was approved by Institutional Animal Care and Use Committee of Ningbo University. The study was conducted in accordance with the local legislation and institutional requirements.

## Author contributions

TD: Investigation, Methodology, Visualization, Writing – original draft. ZZ: Methodology, Writing – review & editing, Investigation, Visualization. TZ: Investigation, Methodology, Visualization, Writing – review & editing. CF: Investigation, Visualization, Writing – review & editing. LN: Methodology, Conceptualization, Funding acquisition, Project administration, Supervision, Writing – review & editing. JC: Writing – review & editing, Conceptualization, Project administration, Supervision.
