# Supplementary material for: The anti-inflammatory role of zDHHC23 through the promotion of macrophage M2 polarization and macrophage necroptosis in large yellow croaker (Larimichthys crocea)
Source: Front Immunol. 2024 May 29;15:1401626. doi: 10.3389/fimmu.2024.1401626 (PMC11167447; doi:10.3389/fimmu.2024.1401626)
Supplement: Supplementary file 1 [file DataSheet_1.pdf]

Supplementary Material

1 Supplementary Figures and Tables

1.1 Supplementary Figures

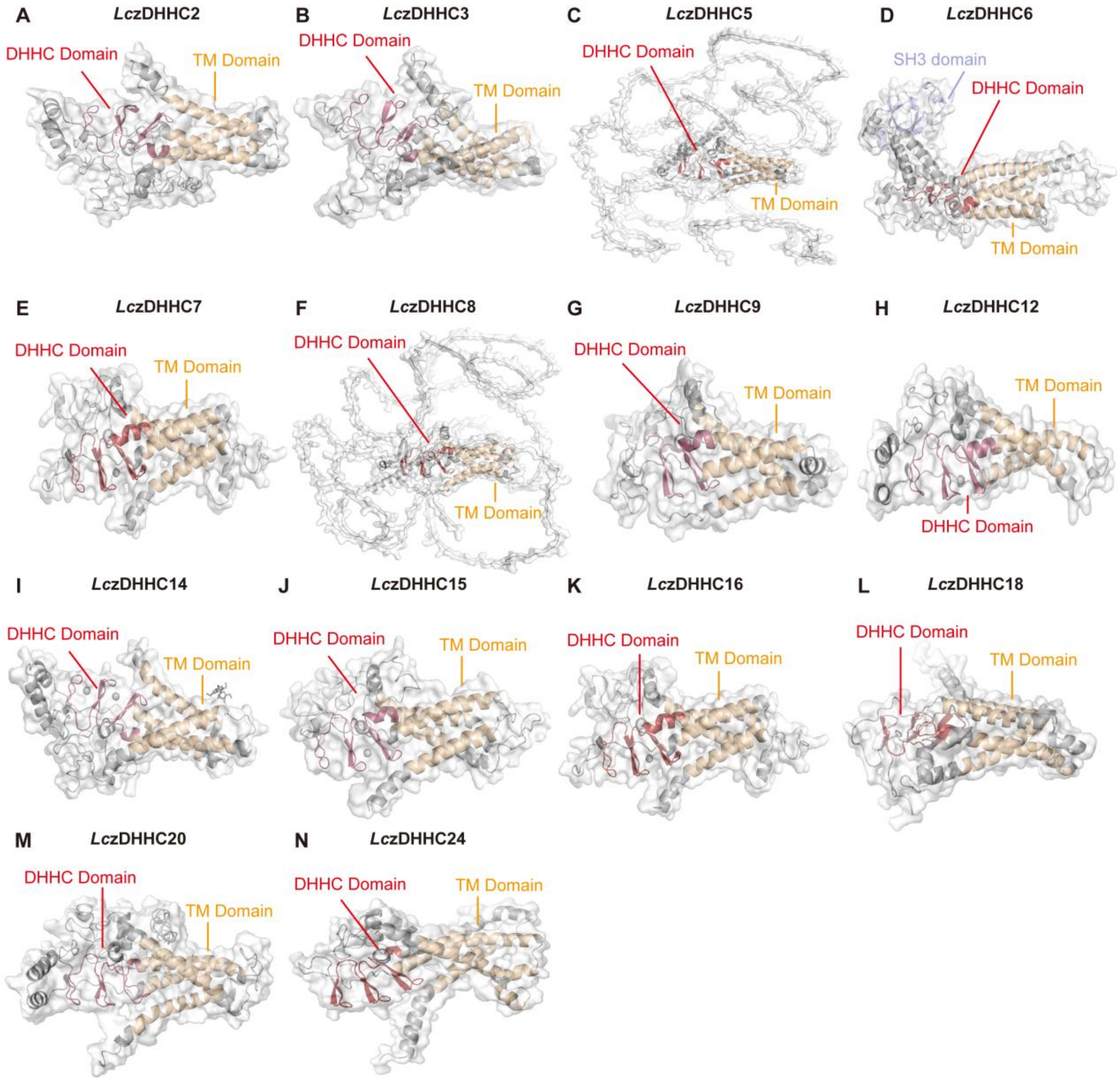

Supplementary Figure 1. Predicted tertiary structures of *Lcz*DHHC family proteins with four transmembrane (TM) domains. The tertiary structures were predicted by SWISS-MODEL and visualized with PyMOL software version 3.1. TM domains are depicted in yellow, the DHHC domain is represented in red, and the SH3 domain is indicated by light blue.

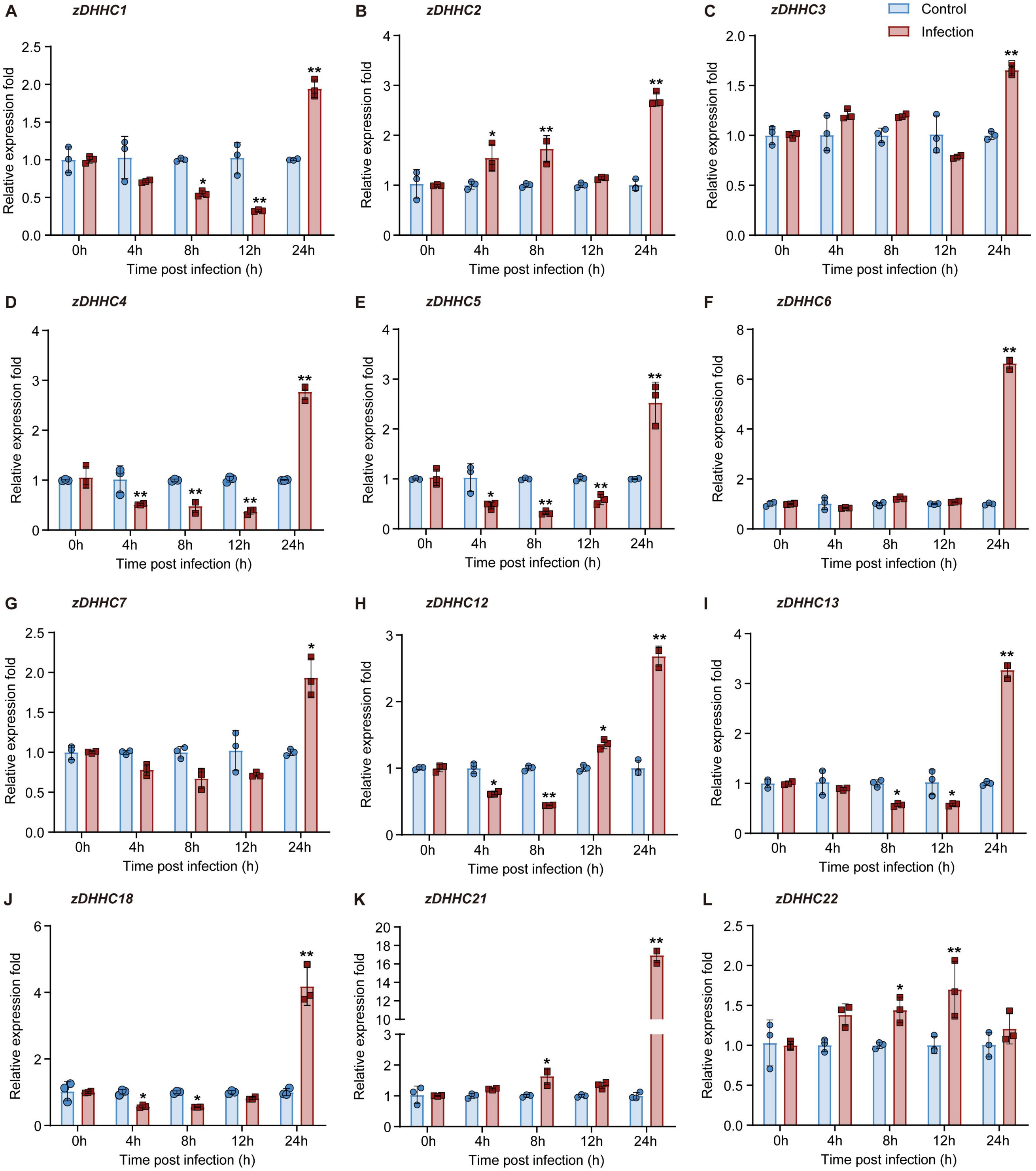

Supplementary Figure 2. Quantitative analysis of *LczDHHCs* whose expression was upregulated in response to *P. plecoglossicida* infection, including *LczDHHC1-7*, *12-13, 18* and *21-22*. MO/MΦ from large yellow croakers were infected with *P. plecoglossicida* at an MOI of 2, with PBS-treated cells as controls. Samples were collected at 0, 4, 8, 12, and 24 hpi. Expression levels of each *LczDHHC* mRNA were normalized to *Lc18S rRNA* and then to the 0 h PBS control using the $2^{-\Delta\Delta CT}$ method. Data represent the means ± SEM of three replicates. **p* < 0.05 and ***p* < 0.01.

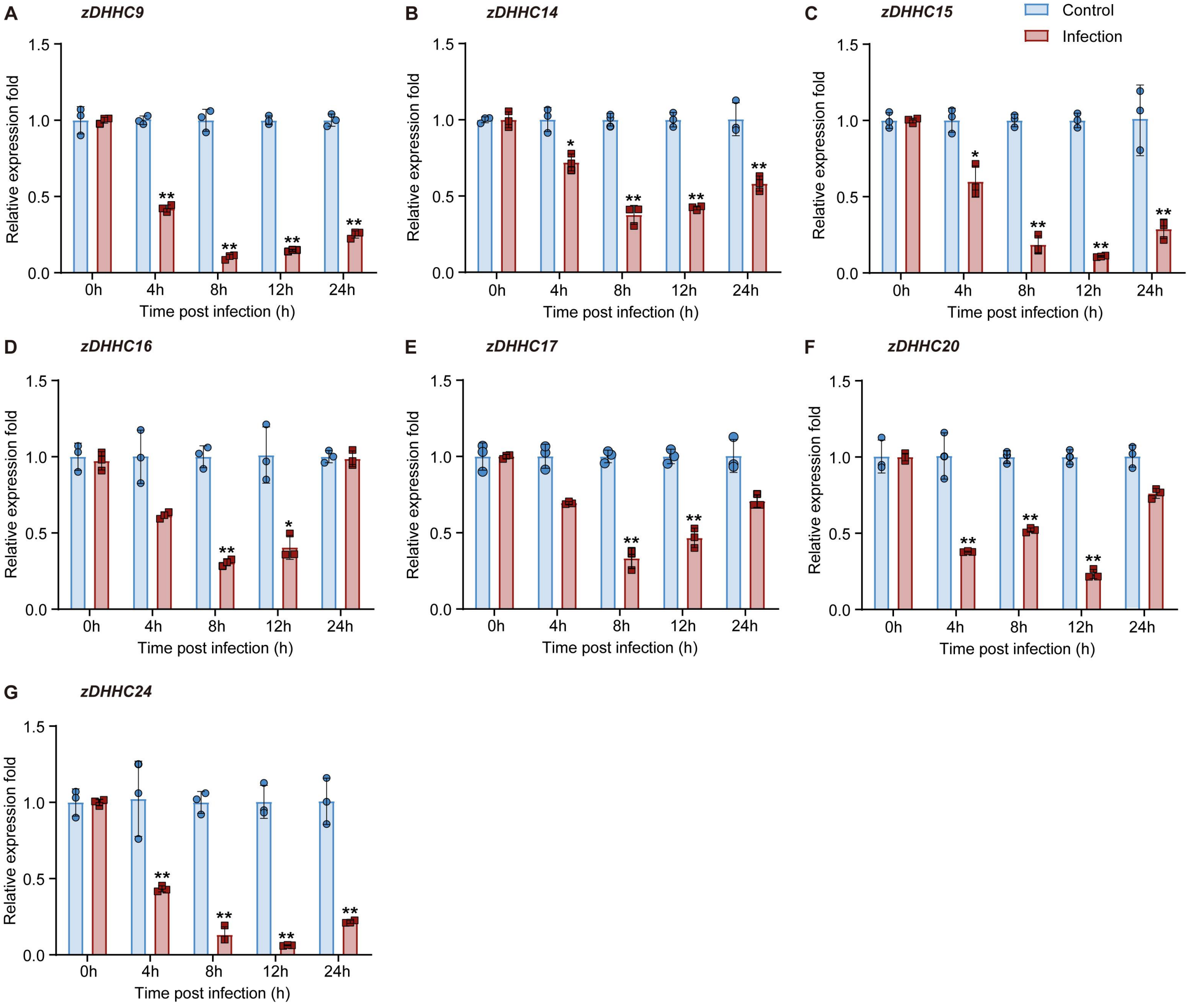

Supplementary Figure 3. Quantitative analysis of *LczDHHCs* whose expression was downregulated in response to *P. plecoglossicida* infection, including *LczDHHC9, 14-15*, *16-17*, *20*, and *24*. MO/MΦ from large yellow croakers were infected with *P. plecoglossicida* at an MOI of 2, with PBS-treated cells as controls. Samples were collected at 0, 4, 8, 12, and 24 hpi. Expression levels of each *LczDHHC* mRNA were normalized to *Lc18S rRNA* and then to the 0 h PBS control using the $2^{-\Delta\Delta CT}$ method. Data represent the means ± SEM of three replicates. $^{*}p < 0.05$ and $^{**}p < 0.01$.

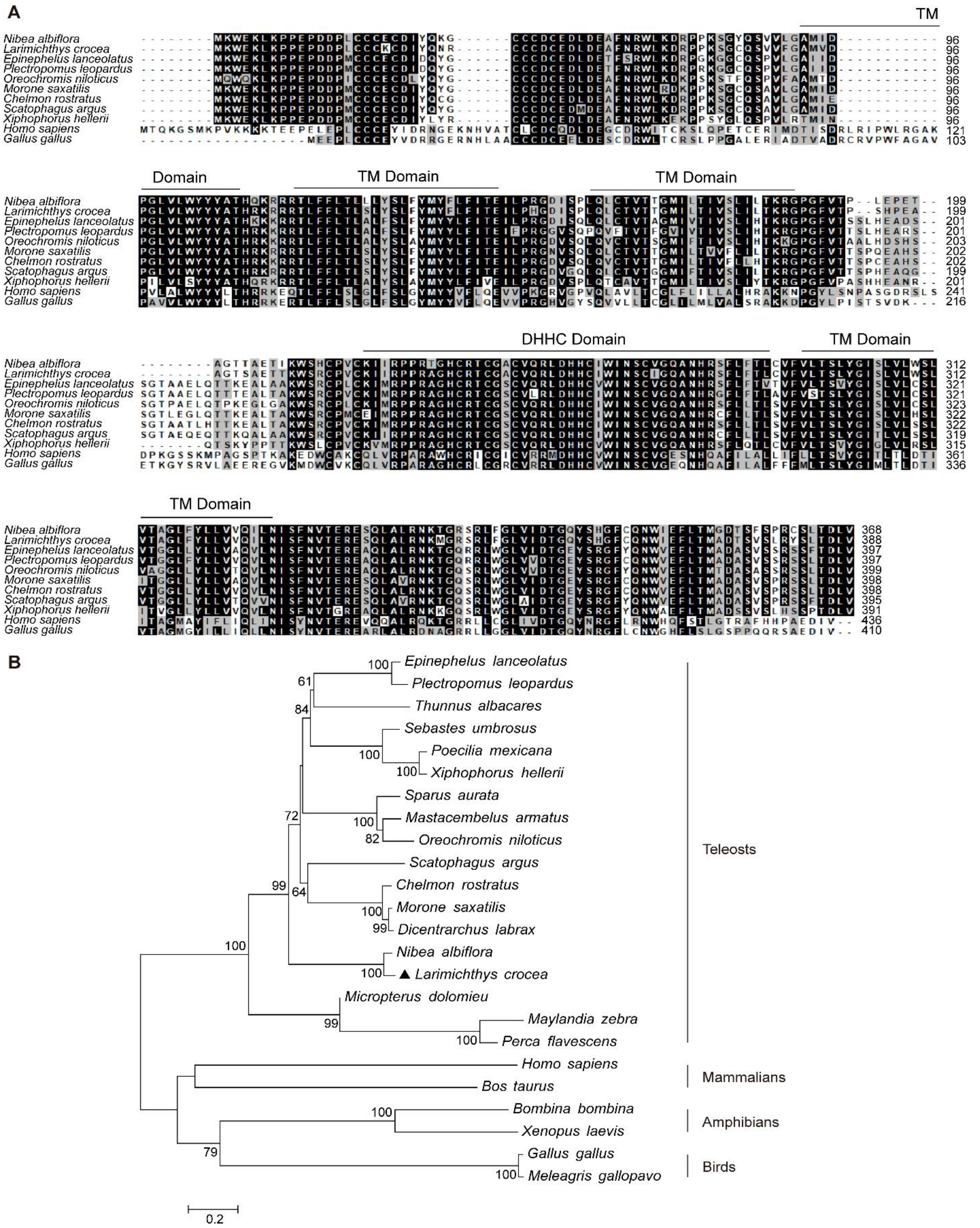

Supplementary Figure 4. Bioinformatics analysis of *Lcz*DHHC23. (A) Multiple sequence alignment of zDHHC23 in teleosts and other species was performed. Similar residues are shaded in gray, identical residues in black, and alignment gaps are represented by "–". The shading threshold is set at

60%. Predicted locations of the TM domain and DHHC domain are illustrated by black lines. GenBank accession numbers of the amino acid sequences are provided in Supplementary Table 3. (B) Phylogenetic tree analysis of *Lc*zDHHC23, along with zDHHC23 homologs from amino acids sequences in teleosts and other species. The values at the forks indicate the percentage of trees in which this grouping occurred after bootstrapping (1000 replicates, shown only when > 60%). The scale bar represents the number of substitutions per base position. GenBank accession numbers of the sequences used are listed in Supplementary Table 3.

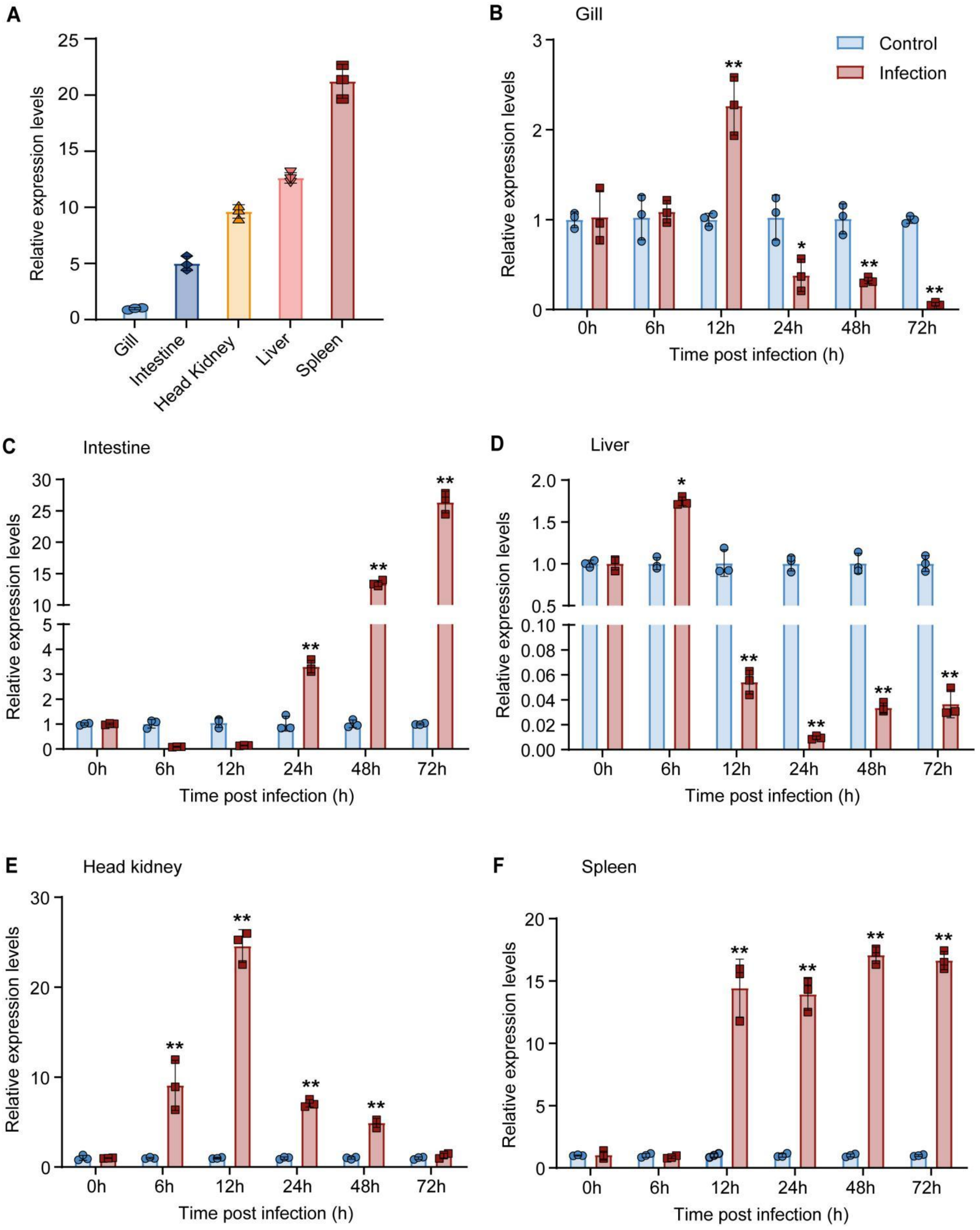

Supplementary Figure 5. The expression pattern of *LczDHHC23* mRNA in large yellow croaker immune tissues before and after *P. plecoglossicida* infection. (A) The expression of *LczDHHC23*

mRNA in healthy large yellow croaker immune tissues (gill, intestine, head kidney, liver, and spleen). (B-F) The expression of *LczDHHC23* mRNA in large yellow croaker challenged with *P. plecoglossicida*. Samples were collected at 0, 4, 8, 12, 24, 48 and 72 hpi, respectively. *LczDHHC23* mRNA expression was normalized to that of *Lc18S rRNA* and then to the 0 h control group using the $2^{-\Delta\Delta CT}$ method. Data represent the means ± SEM of three replicates. $^{*}p < 0.05$ and $^{**}p < 0.01$.

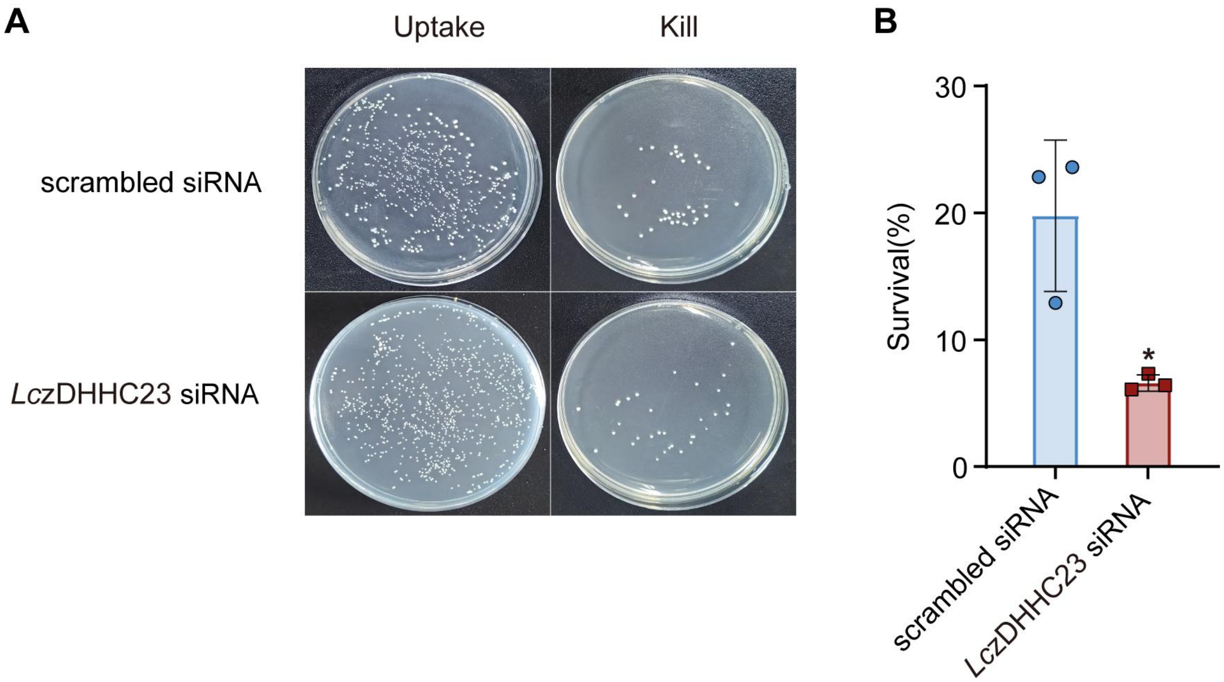

Supplementary Figure 6. Effect of *Lcz*DHHC23 interference on the bactericidal activity of large yellow croaker MO/MΦ. Knocking down *Lcz*DHHC23 enhances the bacterial-killing activity of MO/MΦ. MO/MΦ were transfected with siRNA *Lcz*DHHC23 for 24 h before *P. plecoglossicida* infection at a MOI of 4. The viability of *P. plecoglossicida* was assessed using a colony-forming unit (CFU) assay, based on the plate counting method. Histograms illustrating relative survival rates of *P. plecoglossicida* in both the *Lcz*DHHC23 siRNA and the scrambled siRNA groups were generated. Data represent the means ± SEM of three replicates. *$p < 0.05$ and **$p < 0.01$.

1.2 Supplementary Tables

Supplementary Table 1 Primers used in this study

Primer	Gene	Accession Number	Nucleotide sequence (5’→3’)	Usage
*Lc*zDHHC1-F	*LczDHHC1*	XM_019263266.2	ATGGATGTGTGCAGTAGGAACC	ORF cloning
*Lc*zDHHC1-R			TCACTGCTCCATCCACGA	
*Lc*zDHHC2-F	*LczDHHC2*	XM_010732894.3	ATGGCACCGTCGGGCTCGCGTAGTATCAAGAA	ORF cloning
*Lc*zDHHC2-R			TTAGGCCTCATTTTCTATGGTCATCCCCGG	
*Lc*zDHHC3-F	*LczDHHC3*	XM_027286307.1	ATGAAGAGCCCGGTGCACAG	ORF cloning
*Lc*zDHHC3-R			CTAGTCCAGCGGGATCTCTA	
*Lc*zDHHC4-F	*LczDHHC4*	XM_019252878.2	ATGGATTTCCTCACGCTGTTT	ORF cloning
*Lc*zDHHC4-R			TCAGTTGTCTTTTTTCTGAACAGG	
*Lc*zDHHC5-F	*LczDHHC5*	XM_027285000.1	ATGCCGGGTTTCAGCGGT	ORF cloning
*Lc*zDHHC5-R			TCAAACCGATATCTCGTAAGTGGTCCCTCC	
*Lc*zDHHC6-F	*LczDHHC6*	XM_019264708.2	ATGAACTTCCTGTCAACCTT	ORF cloning
*Lc*zDHHC6-R			TCATAATGGCACTTCTCCAC	

*Lcz*DHHC7-F<br>*Lcz*DHHC7-R	*LczDHHC7*	XM_010734419.3	ATGCAGTCTTCAAACCACAG<br>TCAGACAGAAAACTCAGAGC	ORF cloning
*Lcz*DHHC8-F<br>*Lcz*DHHC8-R	*LczDHHC8*	XM_010733329.3	ATGCCCAACAGCGCTGGAAAGAGATTT<br>TCACACGGATATCTCATATGTGGTGCC	ORF cloning
*Lcz*DHHC9-F<br>*Lcz*DHHC9-R	*LczDHHC9*	XM_019278437.2	ATGTCGGCGGTGATGATAAC<br>CTAATGGGTCTTGTCCTTGG	ORF cloning
*Lcz*DHHC11-F<br>*Lcz*DHHC11-R	*LczDHHC11*	XM_010756920.3	ATGAACTGCTTCAGTCAGA<br>TTAAGTATCATCTGGAGTCATCAC	ORF cloning
*Lcz*DHHC12-F<br>*Lcz*DHHC12-R	*LczDHHC12*	XM_010751980.3	ATGTTCAAAAATGTGTTCGGCTCAGGC<br>CTAGACTTGGTCGCTGCCCTCCCTGAA	ORF cloning
*Lcz*DHHC13-F<br>*Lcz*DHHC13-R	*LczDHHC13*	XM_019253467.2	ATGCACTGGGGCGAAGACGA<br>TCAGACCAGGTCCATGTGCC	ORF cloning
*Lcz*DHHC14-F<br>*Lcz*DHHC14-R	*LczDHHC14*	XM_010738048.3	ATGCATCTCGGGGTGTCTGA<br>TCAGACTGAGCTGAGCTTTACC	ORF cloning

*Lc*zDHHC15-F	*LczDHHC15*	XM_010754621.3	ATGGCTCTCTCCAGAGGTTT	ORF cloning
*Lc*zDHHC15-R			TTACTCTTCCTCCATCTCTATGG	
*Lc*zDHHC16-F	*LczDHHC16*	XM_027289232.1	ATGCGTATGGGCAGCAGC	ORF cloning
*Lc*zDHHC16-R			AATCGATGGCCATCGGGTCAG	
*Lc*zDHHC17-F	*LczDHHC17*	XM_010744348.3	ATGGCCGACGCTATGGAGGA	ORF cloning
*Lc*zDHHC17-R			CTACACCAGCTGGTAGCCTG	
*Lc*zDHHC18-F	*LczDHHC18*	XM_027287289.1	ATGAAAAACTGCGAATATCAGCAAATAGACCC	ORF cloning
*Lc*zDHHC18-R			TCAGTGCGAAGGGGACAAGCT	
*Lc*zDHHC20-F	*LczDHHC20*	XM_019274562.2	ATGGCGCCCATACACGTA	ORF cloning
*Lc*zDHHC20-R			TTAGGACTTGCTCTCCATGG	
*Lc*zDHHC21-F	*LczDHHC21*	XM_010753903.3	ATGAAGTTTCGACTCCACTTTGTGGTGG	ORF cloning
*Lc*zDHHC21-R			CTACACGTGGGTGCGGAAGT	
*Lc*zDHHC22-F	*LczDHHC22*	XM_010733942.3	ATGTTCACCAGGATGTTAAAACTGAGAC	ORF cloning
*Lc*zDHHC22-R			TCAGTCTTGTTTATGTCCATCTGTG	
*Lc*zDHHC2	*LczDHHC23*	XM_01927	ATGGAATCCAGAATGAAATGGG	ORF cloning

3-F		3038.2		
*Lc*zDHHC23-R			CTATACCAAGTCAGTGAGGC	
*Lc*zDHHC24-F	*LczDHHC24*	XM_010749682.3	ATGACGAGTTTAGCCAG	ORF cloning
*Lc*zDHHC24-R			TTACCGGGAAGGTTCGAG	
HA-*Lc*zDHHC23-F	*LczDHHC23*	XM_019273038.2	ATGACGTGCCTGACTATGCCATGGAATCCAGAATGAAATGGG	eukaryotic expression
HA-*Lc*zDHHC23-R			GCCCTCTAGATGCATGCTCGCTATACCAAGTCAGTGAGGC	
Q*Lc*zDHHC1-F	*LczDHHC1*	XM_019263266.2	GTCAGAACCAAGAGTGACAGAG	RT-qPCR
Q*Lc*zDHHC1-R			CTGCAGTGTTTGGACTTAGGA	
Q*Lc*zDHHC2-F	*LczDHHC2*	XM_010732894.3	TCCTCTTCATCGCCCTCATA	RT-qPCR
Q*Lc*zDHHC2-R			GACATGGTACACCAGCAAGTAT	
Q*Lc*zDHHC3-F	*LczDHHC3*	XM_027286307.1	GTAAACGCTGCATACGGAAGA	RT-qPCR
Q*Lc*zDHHC3-R			AGAGTGTGGAGAGAGATGAGTG	
Q*Lc*zDHHC4-F	*LczDHHC4*	XM_019252878.2	CTGGCGGGTTATGCCTTATT	RT-qPCR
Q*Lc*zDHHC4-R			GATGGTCTTCTGTCGCAGTT	

Q*Lcz*DHHC5-F	*LczDHHC5*	XM_027285000.1	GCTATCACTTACCACCCACATC	RT-qPCR
Q*Lcz*DHHC5-R			AGACCAGCCACACACATTAC	
Q*Lcz*DHHC6-F	*LczDHHC6*	XM_019264708.2	CTCCATGAGGTTCGGAGATTG	RT-qPCR
Q*Lcz*DHHC6-R			TAGCGGCCAGTACCAGATAA	
Q*Lcz*DHHC7-F	*LczDHHC7*	XM_010734419.3	TGTTTGGTACACAGATCCACTC	RT-qPCR
Q*Lcz*DHHC7-R			AGATTTCATTCCGTCCCATCTC	
Q*Lcz*DHHC8-F	*LczDHHC8*	XM_010733329.3	CAATGGCCTGGTCTTCCTATTC	RT-qPCR
Q*Lcz*DHHC8-R			CTCGGAAATCATCGTCCTTGTC	
Q*Lcz*DHHC9-F	*LczDHHC9*	XM_019278437.2	AGGTGTTGGTGTGCTTCTT	RT-qPCR
Q*Lcz*DHHC9-R			CCCGACCAGGATCCTTTAATATC	
Q*Lcz*DHHC11-F	*LczDHHC11*	XM_010756920.3	AGTCATGCTGAGCGTCATATC	RT-qPCR
Q*Lcz*DHHC11-R			GTCTCGCTTTCTGGACTCTTT	
Q*Lcz*DHHC12-F	*LczDHHC12*	XM_010751980.3	TGCTCTATTTCGCTGTTTCTCT	RT-qPCR
Q*Lcz*DHHC12-R			GGATTTAGTGGTGGGTGGAAT	

Q*Lcz*DHHC13-F	*LczDHHC13*	XM_019253467.2	CTCTCATGGCTTCAGTCTTCTG	RT-qPCR
Q*Lcz*DHHC13-R			ATGTGCGGAGGTAGTAGTAGAG	
Q*Lcz*DHHC14-F	*LczDHHC14*	XM_010738048.3	GGGAAGGACAACTATAACCCTTAC	RT-qPCR
Q*Lcz*DHHC14-R			CGGTGCGTCTGATTGGATAA	
Q*Lcz*DHHC15-F	*LczDHHC15*	XM_010754621.3	TGATGTTCTCCTGGACCTACT	RT-qPCR
Q*Lcz*DHHC15-R			CCTCTCCTCCATCTCGTATCTT	
Q*Lcz*DHHC16-F	*LczDHHC16*	XM_027289232.1	TCAACAGCCTCAGCAACTC	RT-qPCR
Q*Lcz*DHHC16-R			GAGAGACAAACACCACTCCAA	
Q*Lcz*DHHC17-F	*LczDHHC17*	XM_010744348.3	GTGTCGGAAGTGGGAATCAT	RT-qPCR
Q*Lcz*DHHC17-R			GTGGATCCTCCAGTAGGAAATG	
Q*Lcz*DHHC18-F	*LczDHHC18*	XM_027287289.1	TCACCAACCCATACAGTCATAAA	RT-qPCR
Q*Lcz*DHHC18-R			GATTCATCTGTGGGCAGGAA	
Q*Lcz*DHHC20-F	*LczDHHC20*	XM_019274562.2	TCACCAACCCATACAGTCATAAA	RT-qPCR
Q*Lcz*DHHC20-R			GATTCATCTGTGGGCAGGAA	

Q*Lc*zDHHC21-F	*LczDHHC21*	XM_010753903.3	CTGTGTATCAGCATGGTGTTTG	RT-qPCR
Q*Lc*zDHHC21-R			GATGTGTCCCTCGTTGTAGTG	
Q*Lc*zDHHC22-F	*LczDHHC22*	XM_010733942.3	GTACTCCATCTCCTTTGAGAACC	RT-qPCR
Q*Lc*zDHHC22-R			GCATCAGGACCAGGAACAA	
Q*Lc*zDHHC23-F	*LczDHHC23*	XM_019273038.2	TGTACTGCCCTACCGTCTATAA	RT-qPCR
Q*Lc*zDHHC23-R			ATCTGAACCACCAACAGGTAAA	
Q*Lc*zDHHC24-F	*LczDHHC24*	XM_010749682.3	GGGAAACATCTGCTGGAACT	RT-qPCR
Q*Lc*zDHHC24-R			GAGTGTGTGTCTCGCATGTATAA	
Q*Lc*18S-F	*Lc18S rRNA*	NC_040017	CTCTTAGCTGAGTGTCCCGC	RT-qPCR
Q*Lc*18S-R			ATCTGGCAAGGATCAGCTCA	
Q*Lc*IL-1β-F	*LcIL-1β*	XM_010736551	ATCTGGCAAGGATCAGCTCA	RT-qPCR
Q*Lc*IL-1β-R			ACCAGTTGTTGTAGGGGACG	
Q*Lc*IL-6-F	*LcIL-6*	AOG21085.1	GCCAAGGGCCTGTTCACTTA	RT-qPCR
Q*Lc*IL-6-R			GAGGGCTGACACAAAGACCA	
Q*Lc*IL-10-F	*LcIL-10*	XM_010738826	AGTCGGTTACTTTCTGTGGTG	RT-qPCR
Q*Lc*IL-10-R			TGTATGACGCAATATGGTCTG	
Q*Lc*TGF-β-F	*LcTGF-β*	NW_020851984	GCAACCACCGTACATCCTGA	RT-qPCR

Q*Lc*TGF-β-R			ACCCCATGCAGTAATTGGCA	
Q *Lc*spp1-F	*Lcspp1*	XM_010736988.3	TCCTCAAGCTTCCTCCGACT	RT-qPCR
Q *Lc*spp1-R			TGGAGGTCGTCTCACCATTT	
Q *Lc*cxcl9-F	*Lccxcl9*	XM_019259582.2	TGTGGCTCTGGGTCTTAGTCT	RT-qPCR
Q *Lc*cxcl9-R			GCTGTCTGACTGACTCACTATGAT	
Q *Lc*arg1-F	*Lcarginase1*	XM_019269015.2	GGATCATCGGAGCACCTTTCT	RT-qPCR
Q *Lc*arg1-R			TTGGCTTCACAGCCTTGCTC	
Q*Lc*iNOS-F	*LciNOS*	NC_040032	AGAGAGATCGGGTTCACA	RT-qPCR
Q*Lc*iNOS-R			CACAGAACTGAGGGTACA	

Supplementary Table 2 zDHHC protein family sequences used for phylogenetic tree analysis.

Accession Number	Species		Protein
	Scientific Name	Common Name	
XP_033488147.1	*Epinephelus lanceolatus*	giant grouper	
XP_044052873.1	*Siniperca chuatsi*	mandarin fish	
XP_011485418.1	*Oryzias latipes*	Japanese medaka	zDHHC1
XP_027137031.1	*Larimichthys crocea*	large yellow croaker	
NP_001410714.1	*Danio rerio*	zebrafish	
XP_010731196.2	*Larimichthys crocea*	large yellow croaker	
XP_005460538.1	*Oreochromis niloticus*	Nile tilapia	
XP_032411103.1	*Xiphophorus hellerii*	green swordtail	zDHHC2
XP_017328788.1	*Ictalurus punctatus*	channel catfish	
XP_035505364.1	*Scophthalmus maximus*	turbot	
XP_019119867.1	*Larimichthys crocea*	large yellow croaker	
XP_035477277.1	*Scophthalmus maximus*	turbot	
XP_035536900.1	*Morone saxatilis*	striped sea-bass	zDHHC3
XP_036001234.1	*Fundulus heteroclitus*	mummichog	
XP_044065344.1	*Siniperca chuatsi*	mandarin fish	
XP_019108423.1	*Larimichthys crocea*	large yellow croaker	
XP_033466529.1	*Epinephelus lanceolatus*	giant grouper	zDHHC4
XP_020471563.1	*Monopterus albus*	swamp eel	

XP_039643167.1	*Perca fluviatilis*	European perch	
XP_044034345.1	*Siniperca chuatsi*	mandarin fish	
NP_956343.2	*Danio rerio*	zebrafish	
XP_010752497.1	*Larimichthys crocea*	large yellow croaker	
XP_030280049.1	*Sparus aurata*	gilthead seabream	
XP_033479301.1	*Epinephelus lanceolatus*	giant grouper	zDHHC5
XP_035525523.1	*Morone saxatilis*	striped sea-bass	
XP_044047651.1	*Siniperca chuatsi*	mandarin fish	
XP_019120253.1	*Larimichthys crocea*	large yellow croaker	
XP_044035825.1	*Siniperca chuatsi*	mandarin fish	
XP_035469151.1	*Scophthalmus maximus*	turbot	zDHHC6
NP_001191086.1	*Danio rerio*	zebrafish	
XP_039991267.1	*Xiphias gladius*	swordfish	
XP_027128912.1	*Larimichthys crocea*	large yellow croaker	
XP_021174908.1	*Fundulus heteroclitus*	mummichog	
XP_033485300.1	*Epinephelus lanceolatus*	giant grouper	zDHHC7
XP_053533810.1	*Ictalurus punctatus*	channel catfish	
XP_023809331.1	*Oryzias latipes*	Japanese medaka	
XP_032417047.1	*Xiphophorus hellerii*	green swordtail	
XP_010731631.1	*Larimichthys crocea*	large yellow croaker	zDHHC8

XP_041793002.1	*Chelmon rostratus*	copperband butterflyfish	
XP_046241705.1	*Scatophagus argus*	spotted scat	
XP_030273468.1	*Sparus aurata*	gilthead seabream	
XP_035514049.1	*Morone saxatilis*	striped sea-bass	
NP_840089.3	*Danio rerio*	zebrafish	
XP_019133982.1	*Larimichthys crocea*	large yellow croaker	
XP_035525738.1	*Morone saxatilis*	striped sea-bass	
XP_041653702.1	*Cheilinus undulatus*	humphead wrasse	zDHHC9
XP_030252434.1	*Sparus aurata*	gilthead seabream	
NP_001103496.1	*Danio rerio*	zebrafish	
XP_010755222.2	*Larimichthys crocea*	large yellow croaker	
XP_039662167.1	*Perca fluviatilis*	European perch	zDHHC11
XP_041799138.1	*Chelmon rostratus*	copperband butterflyfish	
XP_035528699.1	*Morone saxatilis*	striped sea-bass	
XP_019127734.1	*Larimichthys crocea*	large yellow croaker	
XP_024659153.1	*Maylandia zebra*	zebra mbuna	
XP_030292188.1	*Sparus aurata*	gilthead seabream	zDHHC12
XP_035524395.1	*Morone saxatilis*	striped sea-bass	
XP_021327289.1	*Danio rerio*	zebrafish	
XP_019109012.2	*Larimichthys crocea*	large yellow croaker	zDHHC13
XP_041789793.1	*Chelmon rostratus*	copperband butterflyfish	

XP_035537574.1	*Morone saxatilis*	striped sea-bass	
XP_033488212.1	*Epinephelus lanceolatus*	giant grouper	
XP_039651118.1	*Perca fluviatilis*	European perch	
NP_001008650.2	*Danio rerio*	zebrafish	
XP_010736349.1	*Larimichthys crocea*	large yellow croaker	
XP_033498329.1	*Larimichthys crocea*	giant grouper	
XP_039642690.1	*Perca fluviatilis*	European perch	zDHHC14
XP_039993094.1	*Xiphias gladius*	swordfish	
NP_001038652.1	*Danio rerio*	zebrafish	
XP_010752923.1	*Larimichthys crocea*	large yellow croaker	
XP_033489795.1	*Epinephelus lanceolatus*	giant grouper	
XP_039669732.1	*Perca fluviatilis*	European perch	zDHHC15
XP_030253303.1	*Sparus aurata*	gilthead seabream	
XP_005467918.1	*Oreochromis niloticus*	Nile tilapia	
NP_001071249.1	*Danio rerio*	zebrafish	
XP_019108877.2	*Larimichthys crocea*	large yellow croaker	
XP_033468008.1	*Epinephelus lanceolatus*	giant grouper	
XP_042253289.1	*Thunnus maccoyii*	southern bluefin tuna	zDHHC16
XP_028423235.1	*Perca fluviatilis*	European perch	
XP_017214210.1	*Danio rerio*	zebrafish	

XP_010742650.1	*Larimichthys crocea*	large yellow croaker	
XP_035534652.1	*Morone saxatilis*	striped sea-bass	
XP_033470673.1	*Epinephelus lanceolatus*	giant grouper	zDHHC17
XP_028426453.1	*Perca fluviatilis*	European perch	
NP_001121854.1	*Danio rerio*	zebrafish	
XP_010752639.3	*Larimichthys crocea*	large yellow croaker	
XP_035489320.1	*Scophthalmus maximus*	turbot	
XP_033488147.1	*Epinephelus lanceolatus*	giant grouper	zDHHC18
XP_042280789.1	*Thunnus maccoyii*	southern bluefin tuna	
XP_028436943.1	*Perca flavescens*	European perch	
NP_001071031.1	*Danio rerio*	zebrafish	
XP_019130107.1	*Larimichthys crocea*	large yellow croaker	
XP_033494993.1	*Epinephelus lanceolatus*	giant grouper	
XP_025766740.1	*Oreochromis niloticus*	Nile tilapia	zDHHC20
XP_042288222.1	*Thunnus maccoyii*	southern bluefin tuna	
XP_036791019.1	*Oncorhynchus mykiss*	rainbow trout	
XP_010752205.2	*Larimichthys crocea*	large yellow croaker	
XP_035530769.1	*Morone saxatilis*	striped sea-bass	
XP_030287717.1	*Sparus aurata*	gilthead seabream	zDHHC21
XP_018546341.1	*Lates calcarifer*	barramundi perch	
NP_001116527.1	*Danio rerio*	zebrafish	

XP_010732244.2	*Larimichthys crocea*	large yellow croaker	
XP_044023037.1	*Siniperca chuatsi*	mandarin fish	zDHHC22
XP_030247771.1	*Sparus aurata*	gilthead seabream	
XP_001340992.2	*Danio rerio*	zebrafish	
XP_019128583.1	*Larimichthys crocea*	large yellow croaker	
XP_030254293.1	*Sparus aurata*	gilthead seabream	
NP_001003757.1	*Danio rerio*	zebrafish	zDHHC23
XP_044225556.1	*Thunnus maccoyii*	southern bluefin tuna	
XP_033507799.1	*Epinephelus lanceolatus*	giant grouper	
XP_010747984.2	*Larimichthys crocea*	large yellow croaker	
XP_035525685.1	*Morone saxatilis*	striped sea-bass	zDHHC24
XP_041800295.1	*Chelmon rostratus*	copperband butterflyfish	
XP_042274755.1	*Thunnus maccoyii*	southern bluefin tuna	

Supplementary Table 3 zDHHC23 sequences used for multiple sequence alignment and phylogenetic tree analysis

Accession Number	Species	
	Scientific Name	Common Name
XP_033507799.1	*Epinephelus lanceolatus*	giant grouper
XP_042355045.1	*Plectropomus leopardus*	leopard coralgrouper
XP_044225556.1	*Thunnus albacares*	southern bluefin tuna
XP_037605717.1	*Sebastes umbrosus*	honeycomb rockfish
XP_014831109.1	*Poecilia mexicana*	shortfin molly
XP_032432443.1	*Xiphophorus hellerii*	green swordtail
XP_030292831.1	*Sparus aurata*	gilthead seabream
XP_026184215.1	*Mastacembelus armatus*	zig-zag eel
XP_003446821.1	*Oreochromis niloticus*	Nile tilapia
XP_046266853.1	*Scatophagus argus*	spotted scat
XP_041808885.1	*Chelmon rostratus*	copperband butterflyfish
XP_035521870.1	*Morone saxatilis*	striped sea-bass
XP_051257022.1	*Dicentrarchus labrax*	European seabass
KAG8008176.1	*Nibea albiflora*	white flower croaker
XP_019128583.1	*Larimichthys crocea*	large yellow croaker
XP_045896108.1	*Micropterus dolomie*	smallmouth bass
XP_004564058.1	*Maylandia zebra*	zebra mbuna

XP_028451796.1	*Perca flavescens*	yellow perch
NP_001307395.1	*Homo sapiens*	human
NP_001179243.1	*Bos taurus*	cattle
XP_053561813.1	*Bombina bombina*	fire-bellied toad
XP_018101310.1	*Xenopus laevis*	African clawed frog
NP_001012837.2	*Gallus gallus*	chicken
XP_003202781.1	*Meleagris gallopavo*	turkey
